# Personality Traits Predict Profiles of Physiological Dysregulation in Adulthood

**DOI:** 10.1093/geroni/igaf122.3382

**Published:** 2025-12-31

**Authors:** Nicholas Turiano, Taylor Brown, Sarah Miller, Meredith Willard

**Affiliations:** West Virginia University, Morgantown, West Virginia, United States; West Virginia University, Morgantown, West Virginia, United States; West Virginia University, Morgantown, West Virginia, United States; West Virginia University, Morgantown, West Virginia, United States

## Abstract

Personality traits are robust predictors of health outcomes across the lifespan, but more evidence is needed how traits specifically impact multiple biological systems. Thus, the current study utilized a sample of 1,239 participants (Mean age = 54.61; range 34-84) from the Midlife Development in the U.S. (MIDUS) study to determine if personality traits would predict extracted latent classes of individuals based on their level of multi-system physiological dysregulation. Personality was assessed with the MIDI Big 5 measure. Twenty-four biomarkers were utilized to create highest quartile proportion scores for each of the 7 different physiological systems measured (para/sympathetic systems, HPA axis, cardiovascular, lipid/glucose metabolism, inflammation). These 7 indicators were used in a latent profile analysis where a 3-class solution provided the best model fit. Multinomial logistic regression analyses revealed that higher levels of conscientiousness significantly predicted (p’s < .05) an increased odds in being in the “lowest” physiological risk class (N = 809) versus the two other classes with clearly more physiological dysregulation. Unique to these two classes was one that had elevated parasympathetic and HPA system dysfunction (N = 228), while the other had less parasympathetic but more HPA dysfunction (N = 217). These findings pinpoint parasympathetic and HPA axis dysfunction s two defining features of physiological health in older adulthood, which is related to level of conscientiousness. Exploring how psychological characteristics are associated with the aging process is key to identifying the adults at-risk of poor health and mortality.

